# Supplementary material for: Factors Associated with the 30-Day and 1-Year Smoking Abstinence of Women in Korea: The Effect of Nicotine Dependency, Self-Efficacy, and Mental Illness
Source: Int J Environ Res Public Health. 2021 Oct 24;18(21):11171. doi: 10.3390/ijerph182111171 (PMC8583369; doi:10.3390/ijerph182111171)
Supplement: Supplementary file 1 [file ijerph-18-11171-s001.zip › ijerph-1398687-supplementary.pdf]

International Journal of
Environmental Research and Public Health

MDPI

Table S1. Odds ratios and 95% confidence intervals of potential factors associated with smoking cessation at the 30-day follow-up including subjects lost to follow up accounted as unsuccessful cessation cases.

Variables	Total [*N*]	Successful Cessation [c] [*N* (%)]	OR (95% CI) [d]	OR (95% CI) [e]
	3,501	818 (23.4)		
Age groups(years)				
≥50	469	110(23.5)	ref	ref
40–-49	657	118(18.0)	0.715(0.534–0.957)	0.776(0.520–1.158)
30–39	1223	307(25.1)	1.094(0.852–1.404)	1.059(0.728–1.540)
20–29	1152	283(24.6)	13063(0.826–1.368)	0.935(0.627–1.396)
p for trend [f]			*p*: 0.0572	*p*: 0.8382
Education level				
Middle school or less	319	66(20.7)	ref	ref
High school	1746	372(21.3)	1.047(0.764–1.436)	1.185(0.783–1.792)
College or more	1375	367(26.7)	1.404(1.014–1.944)	1.450(0.943–2.230)
p for trend [f]			*p*: 0.0019	*p*: 0.0124
Marital status				
Married	1773	394(22.2)	ref	ref
Single	1726	424(24.6)	1.059(0.879–1.277)	1.203(0.951–1.522)
Body mass index (kg/m^2) [a]				
Normal (18.5–22.9)	1994	463(23.2)	ref	ref
Underweight (< 18.5)	557	120(21.5)	0.865(0.688–1.089)	0.831(0.629–1.098)
Overweight or more (≥ 23.0)	937	231(24.7)	1.121(0.932–1.348)	1.237(0.983–1.557)
p for trend [f]			*p*: 0.0580	*p*: 0.0132
Frequency of drinking alcohol per month				
Non-drinker	571	141(24.7)	ref	ref
Less than 5 times	1014	270(26.6)	1.082(0.853–1.373)	0.949(0.732–1.232)
Over 6 times	1189	250(21.0)	0.787(0.619–1.000)	0.757(0.583–0.983)
p for trend [f]			*p*: 0.0118	*p*: 0.0160
Nicotine dependency [b]				
7–10(severe)	552	83(15.0)	ref	ref
4–6(moderate)	1582	319(20.2)	1.422(1.092-1.852)	1.191(0.874-1.623)
0–3(mild)	1303	390(29.9)	2.372(1.822–3.087)	1.730(1.267–2.362)
p for trend [f]			*p*: < 0.0001	*p*: < 0.0001
Self–efficacy for smoking cessation				
0–2	784	91(11.6)	ref	ref
3–6	2027	467(23.0)	2.249(1.766–2.866)	2.102(1.559–2.833)
7–8	591	237(40.1)	5.068(3.853–6.666)	4.736(3.380–6.634)
p for trend [f]			*p*: < 0.0001	*p*: < 0.0001
Presence of quit supporter				
No	986	175(17.8)	ref	ref
Yes	2461	629(25.6)	1.589(1.318–1.916)	1.641(1.312–2.053)
Disease associated with mental				
No	3192	768(24.1)	ref	ref
Yes	200	23(11.5)	0.429(0.275–0.669)	0.390(0.209–0.692)

Note. OR= odds ratios; CI= confidence interval; *p* = *p* for trend. [a] Classification of BMI using the guideline for Asian population20. [b] Fagerstrom Test for nicotine dependence score. [c] Includes participants who maintained cessation for more than 30 days and those who maintained cessation but had not yet completed the 30–day program period. [d] Multiple logistics regression models adjusted for age. [e] Multiple logistics regression models adjusted for age, education level, marital status, BMI, frequency of drinking alcohol per month, nicotine dependency, self-efficacy for smoking cessation, presence of a smoking cessation supporter and presence of a mental illness. [f] Cochran-Armitage trend test.

Table S2. Odds ratios and 95% confidence intervals of potential factors associated with smoking cessation at the 1-year follow-up including subjects lost to follow up accounted as unsuccessful cessation cases.

Variables	Total [*N*]	Successful Cessation [c] [*N* (%)]	OR (95% CI) [d]	OR (95% CI) [e]
	n = 3501	*n* = 255(7.3%)		
Age groups(years)				
≥50	469	30(6.4)	ref	ref
40–49	657	32(4.9)	0.749(0.449–1.251)	0.694(0.348–1.382)
30–39	1223	96(7.9)	1.246(0.815–1.906)	1.117(0.604–2.068)
20–29	1152	97(8.4)	1.345(0.880–2.057)	1.268(0.663–2.425)
p for trend [f]			*p*: 0.0176	*p*: 0.1265
Education level				
Middle school or less	319	17(5.3)	ref	ref
High school	1746	108(6.2)	1.102(0.629–1.931)	1.515(0.708–3.243)
College or more	1375	126(9.2)	1.692(0.958–2.989)	2.192(1.006–4.777)
p for trend [f]			*p*: 0.0040	*p*: 0.0074
Marital status				
Married	1773	121(6.2)	ref	ref
Single	1726	134(7.8)	0.947(0.699–1.283)	0.935(0.647–1.352)
Body mass index (kg/m^2) [a]				
Normal (18.5–22.9)	1994	146(7.3)	ref	ref
Underweight (< 18.5)	557	40(7.2)	0.909(0.630–1.312)	0.915(0.595–1.407)
Overweight or more (≥ 23.0)	937	68(7.3)	1.054(0.778–1.429)	1.191(0.826–1.717)
p for trend [f]			*p*: 0.5211	*p*: 0.2827
Frequency of drinking alcohol per month				
Non–drinker	571	46(8.1)	ref	ref
Less than 5 times	1014	90(8.9)	1.057(0.727–1.537)	0.999(0.675–1.481)
Over 6 times	1189	65(5.5)	0.618(0.415–0.918)	0.620(0.409–0.940)
p for trend [f]			*p*: 0.0049	*p*: 0.0078
Nicotine dependency [b]				
7–10(severe)	552	21(3.8)	ref	ref
4–6(moderate)	1582	92(5.8)	1.540(0.948–2.500)	1.072(0.638–1.800)
0–3(mild)	1303	137(10.5)	2.848(1.775–4.570)	1.547(0.929–2.576)
p for trend [f]			*p*: < 0.0001	*p*: 0.0173
Self–efficacy for smoking cessation				
0–2	784	26(3.3)	ref	ref
3–6	2027	143(7.1)	2.160(1.409–3.309)	1.959(1.171–3.279)
7–8	591	78(13.2)	4.367(2.762–6.907)	3.660(2.097–6.389)
p for trend [f]			*p*: < 0.0001	*p*: < 0.0001
Presence of quit supporter				
No	986	55(5.6)	ref	ref
Yes	2461	199(8.1)	1.480(1.087–2.015)	1.256(0.885–1.784)
Disease associated with mental				
No	3192	248(7.8)	ref	ref
Yes	200	6(3.0)	0.401(0.176–0.916)	0.212(0.052–0.873)

Note. OR= odds ratios; CI= confidence interval; *p* = *p* for trend. [a] Classification of BMI using guideline for Asian population20-Pacific. [b] Fagerstrom Test for nicotine dependence score. [c] Includes participants who maintained cessation for more than one year and those who maintained cessation but had not yet completed the 1-year program period. [d] Multiple logistics regression models adjusted for age. [e] Multiple logistics regression models adjusted for age, education level, marital status, BMI, frequency of drinking alcohol per month, nicotine dependency, self-efficacy for smoking cessation, presence of a smoking cessation supporter, and presence of a mental illness. [f] Cochran-Armitage trend test.
